# A serological assay to detect and differentiate rodent exposure to soft tick and hard tick relapsing fever infections in the United States

**DOI:** 10.1016/j.ttbdis.2023.102167

**Published:** 2023-03-23

**Authors:** Christina M. Parise, Ying Bai, Kevin S. Brandt, Shelby L. Ford, Sarah Maes, Adam J. Replogle, Alexander R. Kneubehl, Job E. Lopez, Rebecca J. Eisen, Andrias Hojgaard

**Affiliations:** a Division of Vector-Borne Diseases, National Center for Emerging and Zoonotic Infectious Diseases, Centers for Disease Control and Prevention, 3156 Rampart Road, Fort Collins, CO 80521, USA; b Department of Pediatrics, National School of Tropical Medicine at Baylor College of Medicine One Baylor Plaza, BCM113, Houston, TX 77030, USA; c Department of Pediatrics and Molecular Virology and Microbiology, National School of Tropical Medicine at Baylor College of Medicine One Baylor Plaza, BCM113, Houston, TX 77030, USA

**Keywords:** Relapsing fever, Borrelia, Recombinant proteins, Serology

## Abstract

Human cases of relapsing fever (RF) in North America are caused primarily by *Borrelia hermsii* and *Borrelia turicatae*, which are spread by argasid (soft) ticks, and by *Borrelia miyamotoi*, which is transmitted by ixodid (hard) ticks. In some regions of the United States, the ranges of the hard and soft tick RF species are known to overlap; in many areas, recorded ranges of RF spirochetes overlap with Lyme disease (LD) group *Borrelia* spirochetes. Identification of RF clusters or cases detected in unusual geographic localities might prompt public health agencies to investigate environmental exposures, enabling prevention of additional cases through locally targeted mitigation. However, exposure risks and mitigation strategies differ among hard and soft tick RF, prompting a need for additional diagnostic strategies that differentiate hard tick from soft tick RF. We evaluated the ability of new and previously described recombinant antigens in serological assays to differentiate among prior exposures in mice to LD, soft or hard tick RF spirochetes. We extracted whole-cell protein lysates from RF *Borrelia* cultures and synthesized six recombinant RF antigens (*Borrelia* immunogenic protein A (BipA) derived from four species of RF *Borrelia*, glycerophosphodiester phosphodiesterase (GlpQ), and *Borrelia miyamotoi* membrane antigen A (BmaA)) to detect reactivity in laboratory derived (*Peromyscus* sp. and *Mus* sp.) mouse serum infected with RF and LD *Borrelia* species. Among 44 *Borrelia* exposed mouse samples tested, all five mice exposed to LD spirochetes were correctly differentiated from the 39 mice exposed to RF *Borrelia* using the recombinant targets. Of the 39 mice exposed to RF spirochetes, 28 were accurately categorized to species of exposure (71%). Segregation among soft tick RF species (*Borrelia hermsii, Borrelia parkeri* and *Borrelia turicatae*) was inadequate (58%) owing to observed cross-reactivity among recombinant BipA protein targets. However, among the 28 samples accurately separated to species, all were accurately assigned to soft tick or hard tick RF type. Although not adequately specific to accurately categorize exposure to soft tick RF species, the recombinant BipA protein targets from soft and hard tick RF species show utility in accurately discriminating mouse exposures to LD or RF *Borrelia*, and accurately segregate hard tick from soft tick RF *Borrelia* exposure.

## 1. Introduction

In the United States, relapsing fever (RF) infections in humans are caused by *Borrelia hermsii, Borrelia turicatae* (both agents of soft tick relapsing fever) and *Borrelia miyamotoi* (hard tick relapsing fever). *Borrelia parkeri* and *Candidatus* Borrelia johnsonii have been implicated as agents of human disease (Davis et al., 1939; Kingry et al., 2018). Clinical management is similar across tick acquired human RF infections (Hasin et al., 2006; Krause and Barbour, 2015; Wagemakers et al., 2015), although neurotropism is more common in *B. miyamotoi* and *B. turicatae* cases and requires specialized care (Lopez et al., 2016). Exposure risks and mitigation strategies differ among ixodid (hard tick) and argasid (soft tick) species, due to the differences in ecologies of the vector ticks. For example, *B. hermsii* is transmitted by soft ticks (*Ornithodoros hermsi*), with human exposures often associated with rustic, rodent-infested cabin settings in the Rocky Mountain and Sierra-Nevada Mountain regions at higher elevations (Thompson et al., 1969; Dworkin et al., 2002). *Borrelia turicatae* is also transmitted by soft ticks (*Ornithodoros turicata*), however this species is more commonly encountered in areas of lower elevation, in cave settings, and burrows in the south-central U.S (Dworkin et al., 2002). By contrast, *B. miyamotoi* is transmitted by hard ticks: blacklegged ticks (*Ixodes scapularis*) and Western blacklegged ticks (*Ixodes pacificus*) in woodlands in the eastern and far western United States, respectively (Scoles et al., 2001; Mun et al., 2006; Fleshman et al., 2022). Prevention methods for tick-acquired human RF cases typically focus on reducing soft tick exposure in dwellings (rodent and tick elimination) for *O. hermsi* (Paul et al., 2002), avoidance of risk settings (infested caves or burrows for *O. turicata*, infested woodlands for *I. scapularis* or *I. pacificus*) or use of effective repellents to avoid tick bites (Mehr et al., 1986).

There are two primary approaches to detect endemic foci of RF spirochetes, direct and indirect detection of the pathogens. Direct detection of infected ticks in presumptive exposure sites indicates on-going enzootic transmission of RF spirochetes. Although this method is the ideal way to confirm an area of human exposure risk, the collection of soft ticks can often be challenging or infeasible due to time constraints (Paul et al., 2002), or property owner’s willingness to allow retrieval of rodent nests and associated soft ticks (a process that can be intrusive or destructive). Alternatively, investigators may rely on the direct detection of the pathogen from rodents to confirm an area of potential exposure (Jones et al., 2016). Because of the relapsing presentation in rodents leading to inconsistent spirochete loads circulating in the blood, traditional direct detection methods of qPCR or microscopy (Boyle et al., 2014; Lynn et al., 2019; Burgdorfer et al., 1970) may yield false negative results if blood is not obtained during a relapse period. For example, previous work by Johnson et al. (2016) demonstrated that only 2.9% of animals had spirochetes detectable by microscopy at the time of sampling, but 27.5% of animals were seropositive for RF antigens. Moreover, the number of animals sampled during this work (n=666) over the course of multiple years of investigation was much higher than would be sampled from during a typical environmental investigation following a human case.

If ticks are unable to be collected and tested during an environmental investigation, the indirect detection of RF spirochete exposures in wildlife by the utilization of serological assays is relied upon (Armstrong et al., 2018; Johnson et al., 2016; Fritz et al., 2013, Fritz et al., 2004; Schwan et al., 2009). However, such assays that can differentiate hard tick from soft tick RF exposures are needed. A glycerophosphodiester phosphodiesterase (GlpQ) protein (Schwan et al., 1996) can differentiate RF antibodies from Lyme disease (LD) associated antibodies; the gene is known to be highly conserved among RF *Borrelia* species (Lopez et al., 2016). Additional protein antigens that have been identified which have demonstrated higher levels of species-specificity among RF *Borrelia* are *Borrelia* immunogenic protein A (BipA) (Lopez et al., 2010, 2013; Curtis et al., 2022) and *Borrelia miyamotoi* membrane antigen A (BmaA) (Harris et al., 2020). Here, we synthesized these previously characterized RF recombinant targets in addition to identifying and synthesizing a BipA homolog target in *B. miyamotoi*. We evaluated these targets by assessing reactivity with serum derived from mice experimentally infected with RF or LD *Borrelia* species to detect and differentiate LD antibodies, hard, and soft tick RF antibodies in mice.

## 2. Materials and methods

### 2.1. Bacterial isolates and growth conditions

To evaluate experimental mouse exposure to RF *Borrelia* by serology, we created whole-cell protein lysates from *B. hermsii, B. parkeri, B. turicatae* and *B. miyamotoi*. Low passage (<10 media passages) starting cultures of all *Borrelia* species were used. *Borrelia hermsii* NE95- 0544 (Trevejo et al., 1998), *B. turicatae* TX15–4654 (BTE5EL; Christensen et al., 2017), *B. parkeri* CA216 (Schwan et al., 2007), and *B. miyamotoi* CT13–2396 (Kingry et al., 2017a) were obtained from the Centers for Disease Control and Prevention reference collection. *Borrelia hermsii, B. turicatae, B. parkeri, B. miyamotoi* CT13–2396 were grown at 34 °C in Barbour-Stoenner-Kelly (BSK-R: *B. miyamotoi* and BSK-IIB: other RF species) media formulations described in Replogle et al. (2021). Dark-field microscopy and a Cellometer counting chamber (Electron Microscopy Sciences, Hatfield, PA, USA) were used to quantify spirochete concentrations in the cultures.

### 2.2. Generation of whole-cell protein lysate from Borrelia spp.

Whole-cell protein lysates were generated for use as antigens in our serological assays. *Borrelia hermsii, B. turicatae*, and *B. parkeri* were grown for 9–15 days to an average of 1.35×10^8^ spirochetes/mL and pelleted *via* centrifugation at 3000 × g for 20 min. *Borrelia miyamotoi* CT13–2396 was grown for 10 days to a density of 8.59×10^7^ spirochetes/mL and pelleted at 8000 × g for 20 min. The supernatant was decanted, and cell pellets were resuspended and washed twice (1x Phosphate Buffered Saline, 5 mM MgCl_2_, pH 7.4), then stored at −80 °C prior to sonication. Pellets were resuspended (1x Tris 10 mM, EDTA 1 mM pH 8.0) with 1 EDTA-free cOmplete Protease Inhibitor tablet (Roche Applied Sciences, Indianapolis, IN, USA). Individual cell pellets from *B. turicatae, B. hermsii, B. parkeri*, and *B. miyamotoi* were sonicated on ice using a Fisher Sonifier 120 (Fisher Scientific, Pittsburgh, PA, USA) at 50% amplitude with a 15 s pulse and 30 s off time (total of 2 min sonication time) followed by 30 s on and 30 s off (total of 1 min 30 s sonication time). A Pierce bicinchoninic acid (BCA) protein assay kit (Thermo Fisher Scientific, Waltham, MA, USA) was used to determine the concentration of the resulting protein lysate according to manufactures protocol.

### 2.3. Cloning, transformation, and expression of recombinant RF proteins in Escherichia coli

We identified the potential antigenic proteins from RF *Borrelia* genomes available on GenBank to obtain the recombinant targets for use in this assay. Proteins were selected from *B. miyamotoi* LB-2001, *B. turicatae* 91E135, *B. parkeri* HR1, and *B. hermsii* DAH genomes. We selected the hypothetical protein (GenBank: WP_070401628.1) on the *B. miyamotoi* LB-2001 plasmid lp72 as a BipA homolog in *B. miyamotoi*, in addition to a GlpQ protein (GenBank: AAG24363.1) in *B. turicatae* 91E135 to clone. The gene sequences for the hypothetical protein identified in *B. miyamotoi* were compared between isolates LB-2001 and CT13–2396 (also located on plasmid lp72 in CT13–2396). The amino acid sequences for BipA proteins from *B. turicatae* 91E135 BipA (GenBank: ADN26518.1), *B. parkeri* HR1 BipA (GenBank: AHF45615.1), and *B. hermsii* DAH BipA (GenBank: ACS27065.1) were compared with the sequence for the *B. miyamotoi* LB-2001 BipA homolog. Gene and protein sequence alignments were performed using Lasergene (DNASTAR, Madison, WI) MegAlign Pro 17 software, and analyzed using the ClustalW algorithm.

To produce these protein targets, we transformed the genes for all the required proteins into *Escherichia coli* for expression. The *bmaA* gene from *B. miyamotoi* LB-2001 was cloned and transformed into *E. coli* BL21 (DE3) (Lucigen, Middleton, WI, USA) as described in Harris et al. (2019). The *bipA* genes from *B. turicatae* 91E135, *B. parkeri* HR1, and *B. hermsii* DAH were transformed into *E. coli* BL21 (DE3) (Thermo Fisher Scientific) as previously described in Curtis et al. (2022). The genes for *B. turicatae glpQ* (GenBank: AF247157.1) and *B. miyamotoi bipA* (GenBank locus tag: bmLB2001_RS05095; signal peptide not included) were amplified using the primers listed in Table 1 using polymerase chain reaction (PCR). The PCR products were cloned into a pETite N-His vector and transformed into *E. coli* BL21 (DE3) (Lucigen) using the Expresso T7 cloning kit (Lucigen). Growth and induction of all transformed clones using IPTG (Thermo Fisher Scientific) was carried out according to the recommendations from each *E. coli* manufacturer (Thermo Fisher Scientific and Lucigen). Individual induced cultures containing each of the recombinant targets were pelleted at 4000 × g for 30 min, the supernatant decanted, and stored at −80 °C until purification. One unit of benzonase nuclease (Millipore Sigma, Burlington, MA, USA) per milliliter of culture and 1 EDTA-free cOmplete protease inhibitor tablet (Roche Applied Sciences, Indianapolis, IN) was added to the pellet. All recombinant proteins were purified using the QiaExpress Ni-NTA FastStart kit (Qiagen, Valencia, CA, USA) under denaturing conditions according to the manufacturer’s instructions. A Pierce BCA protein assay kit (Thermo Fisher Scientific) was used to determine the concentration of the eluted protein.

### 2.4. Laboratory exposure of mice to B. hermsii, B. parkeri, B. turicatae, B. miyamotoi, B. mayonii, and B. burgdorferi s. s. and generation of anti-B. miyamotoi BipA serum

To test the ability of our assay to differentiate host exposure from hard tick vs. soft tick RF *Borrelia* species, we generated mouse immune serum by exposing animals to one of four different species of RF *Borrelia* (*B. hermsii, B. parkeri, B. turicatae*, and *B. miyamotoi*) to generate immune serum. Since *Peromyscus* spp. are natural hosts for tick species that vector RF *Borrelia* (Barbour et al., 2009; Johnson et al., 2016; Salkeld et al., 2018; Armstrong et al., 2018) and are often trapped and sampled for anti-*Borrelia* antibodies, we included *Peromyscus maniculatus* (Peromyscus Genetic Stock Center, Columbia, SC) in addition to *Mus musculus* mice (Charles River Laboratories, Wilmington, MA) in our experiment. Additionally, *Peromyscus leucopus* mice (Peromyscus Genetic Stock Center) infected with *Borrelia mayonii* (Parise et al., 2020) and *M. musculus* mice infected with *Borrelia burgdorferi* sensu stricto (Breuner et al., 2020) were included to evaluate specificity of the recombinant targets for RF *Borrelia* species compared to LD *Borrelia* species. Different *Borrelia* isolates from those which the recombinant antigens originated (listed in previous section) were used to immunize mice against *B. miyamotoi, B. parkeri, B. turicatae*, and *B. hermsii*, because these live RF isolates were available to us, although we reasoned this should be an effective method of generating anti-RF serum as previously demonstrated in Curtis et al. (2022).

All mice used as a part of this study were inoculated subcutaneously with 100 μl 1 × 10^5^ spirochetes. Female 2–7-month-old *P. maniculatus* mice (Peromyscus Genetic Stock Center) were inoculated with *B. hermsii* NE95–0544*, B. turicatae* TX15–4645, *B. parkeri* CA216, or *B. miyamotoi* CT13–2396. Female one-to three-month-old, outbred CD-1 *M. musculus* mice (Charles River Laboratories) were also inoculated with the same isolates as *P. maniculatus*. Mice were bled at 4-and 15-days post infection by submandibular vein puncture, and 100 μl of whole blood was collected. Mice were exsanguinated at 12 weeks post inoculation, and serum was collected and stored at −80 °C.

Serum samples from other *Borrelia* species were also used in this study. Serum from two *M. musculus* mice (Charles River Laboratories) infected with *B. burgdorferi* s.s. B31 *via I. scapularis* using methods described in Breuner et al. (2020) was collected at 4 weeks post exposure. Serum derived from previous work described in Breuner et al. (2017) and Parise et al. (2020) from 13 *M. musculus* mice (Charles River Laboratories) infected with *B. miyamotoi* CT15–0840 *via I. scapularis* and 3 *P. leucopus* mice (Peromyscus Genetic Stock Center) infected with *B. mayonii* MN17–4755 *via I. scapularis* was collected at 8–12 weeks post exposure. Control serum collected from three *P. maniculatus* (Peromyscus Genetic Stock Center)*, P. leucopus* (Peromyscus Genetic Stock Center), and *M. musculus* mice (Charles River Laboratories) of each species with no history of *Borrelia* exposure was included to assess background reactivity to inherent mouse antibodies.

To assess the immunogenicity of recombinant *B. miyamotoi* BipA, three CD1 *M. musculus* (Charles River Laboratories) mice were immunized with the recombinant protein combined with adjuvant and anti-*B. miyamotoi* BipA serum was generated. A primary inoculation of 16 μg of *B. miyamotoi* rBipA that was purified to remove endotoxin (Brandt et al., 2014) and combined with Imject adjuvant were given, followed by two booster inoculations of 16 μg and 12 μg of *B. miyamotoi* rBipA 3 weeks apart. Mice were bled 8–14 days after the last boost. The anti-*B. miyamotoi* BipA serum generated from these mice was used to probe blots with recombinant antigens as previously described.

Animal use and experimental procedures were conducted in accordance with approved protocols on file with the Centers for Disease Control and Prevention Division of Vector-Borne Diseases Animal Care and Use Committee.

### 2.5. Confirmation of Borrelia infection in laboratory exposed mice using PCR or ear tissue culturing

Infection confirmation of laboratory exposed mice was completed by collecting blood or ear tissue samples and detection of *Borrelia* by real-time TaqMan PCR, or culturing. Whole blood samples were collected from the mice as described in Section 2.4, and DNA was extracted following methods in Lynn et al. (2019). PCR reactions were formulated using in-house master mixes that included primers and probes for either of the following DNA targets: a GAPDH target included in the TaqMan Rodent GAPDH ControlReagents kit (ThermoFisher Scientific), or a pan *Borrelia* 16S target (Kingry et al., 2017b) with primers: 16S rDNA-F AGCYTT-TAAAGCTTCGCTTGTAG, 16S rDNA-R GCCTCCCGTAGGAGTCTGG, 16S rDNA-probe HEX-CCGGCCTGAGAGGGTGAWCGG-BHQ1. The reactions were performed in 15 μl solutions with 7.5 μl 2x iQ Multiplex Powermix (Bio-Rad, Hercules, CA, USA), 5 μl DNA extract, and 2.5 μl PCR primers (1.8 μm) and probes (1.2 μm) resuspended in PCR grade water. Cycling conditions comprised an initial 3-min activation step at 95 °C followed by 40 cycles of 95 °C for 10 s and 60 °C for 45 s. We analyzed samples using CFX Manager 3.1 software (Bio-Rad) with the quantitation cycle (Cq) determination mode set to regression. Based on the criteria in Graham et al. (2018), only Cq values<40 was considered positive for the presence of *Borrelia* DNA in the sample.

Mice were confirmed infected with *B. mayonii* or *B. burgdorferi* s.s. by culturing ear tissue biopsies taken from mice at 3 weeks after nymphal tick infestation by using methods described in Parise et al. (2020) and Breuner et al. (2020). Mice infected with *B. miyamotoi* CT15–0840 were bled 9–11 days following nymphal tick infestation, DNA was extracted, and samples were tested by PCR following methods described in Breuner et al. (2017). Only mice with confirmed spirochete infection by detection of *Borrelia* in blood or ear tissue by PCR or culture were included in this study aside from negative control samples (Table 2).

### 2.6. Antigen preparation, SDS-PAGE, and immunoblotting

Western blotting of membranes impregnated with the recombinant protein targets was utilized to test the serum generated from experimentally derived *Borrelia* infected mice for reactivity. The recombinant proteins were separated by size using gel electrophoresis and the resulting gels were either transferred to a nitrocellulose membrane or stained. An EZ-Run Prestained Rec Protein Ladder (Fisher Scientific) molecular weight marker was loaded at 5 μl to identify protein separation by size. Each antigen, at 0.3 μg, was loaded individually or pooled to be separated by gel electrophoresis. All antigens were added to 1x NuPAGE LDS sample buffer (Invitrogen, Carlsbad, CA, USA), 1x NuPAGE sample reducing agent (Invitrogen) and ultra-pure water to a 10 μl final loading volume. Samples were heated at 70° C for 10 min, then vortexed and spun briefly and loaded into the lane. Proteins were electrophoresed in mini pre-cast 15-well 4–12% Bis-Tris NuPAGE gels 1.0 mm (Thermo Fisher Scientific) at 200 V for 52 min using a Surelock Mini Cell (Thermo Fisher Scientific) with 1x NuPAGE MOPS SDS running buffer (Thermo Fisher Scientific). Coomassie staining using SimplyBlue SafeStain (Invitrogen) and transference to nitrocellulose membranes using an iBlot2 Gel Transfer Device (Invitrogen) were completed according to the manufacturer’s instructions. Protein impregnated membranes were stored at 4 °C until serum testing.

Blots were blocked with SuperBlock T20 (TBS) blocking buffer (Thermo Fisher Scientific) for 30 min. Primary antibodies were applied to the membrane for serum samples in a 1:200 dilution, or in a 1:000 dilution for the 6x His monoclonal antibody (Clonetech, San Jose, CA), and incubated for 1 h. Blots were washed with 1x TBS 0.5% Tween 20 (Boston BioProducts, Milford, MA, USA) three times for 5 min, then incubated with Pierce recombinant protein A/G, alkaline phosphatase conjugated (Thermo Fisher Scientific) in a 1:5000 dilution for 30 min. Blots were washed again with 1x TBS 0.5% Tween 20 (Boston BioProducts) three times for 5 min, washed with de-ionized water for 1 min once, then developed using 1-Step NBT/BCIP substrate solution (Thermo Fisher Scientific) for 5 min. Blots were washed with de-ionized water three times and dried on Whatman chromatography paper (Cytiva, Marlborough, MA, USA). Stained gels and blots were imaged using a ChemiDoc imager (Bio-Rad) and ImageLab software (Bio-Rad) using protein gel settings for Coomassie images, or colorimetric blot settings set to intense bands for western blot images.

### 2.7. Assessment of mouse seroreactivity to whole-cell lysate and recombinant protein targets

Each blot was evaluated by three raters that were blinded to the exposure history of the serum sample and given instructions for the scoring scheme resulting in pathogen calls. The final assessment for each sample was designated by the majority call among raters. This procedure was included to ensure rater objectivity and reproducibility when classifying a *Borrelia* positive sample as hard vs. soft tick RF.

Seroconversion to protein whole-cell lysate was assessed as previously described by Armstrong et al. (2018), which required the appearance of at least five bands by Western blot as evidence of exposure to a *Borrelia* species. The whole-cell lysate is broadly reactive across *Borrelia* species and therefore cannot discriminate between antibodies from RF or LD species (Krause et al., 2018). A sample with only reactivity to the whole-cell lysate and none of the recombinant proteins was categorized as positive for *Borrelia*, but inconclusive for species identification. A positive call for *Borrelia* on the whole-cell lysate was the initial screening criteria for further classification of reactivity with recombinant RF targets. A negative call (less than 5 bands on the whole-cell lysate) would result in no further evaluation of the sample.

Recombinant BipA antigens from different RF spirochete species were used to determine the species or type (hard or soft tick) of RF causing infection. For a sample to be considered positive for a soft tick RF, it was required to be positive for a single rBipA target from *B. hermsii, B. parkeri*, or *B. turicatae* and not reactive with *B. miyamotoi* rBipA. For a sample to be considered positive for hard tick RF, it must be positive for *B. miyamotoi* rBipA and not reactive with any of the soft tick RF rBipA targets. In the event of reactivity present towards rBipA targets from more than one *Borrelia* species, cross reactivity was assumed and the sample was called positive for *Borrelia*, but inconclusive for the species. The expected reactivity with each rBipA target is summarized for each pathogen in Table 3. Serum reactivity with rGlpQ and rBmaA targets was also assessed and data is presented in Table 4. Although reactivity with these targets would be considered confirmatory of exposure to a RF spirochete, reactivity with these targets did not affect the pathogen call of the sample in this analysis. The rGlpQ target was predicted to be reactive with serum from mice infected with RF *Borrelia* species and not LD *Borrelia* species, and rBmaA was predicted to be reactive with mouse serum infected with hard tick RF *Borrelia* only.

## 3. Results

### 3.1. Characterization of recombinant antigens

**Fig. 1.**
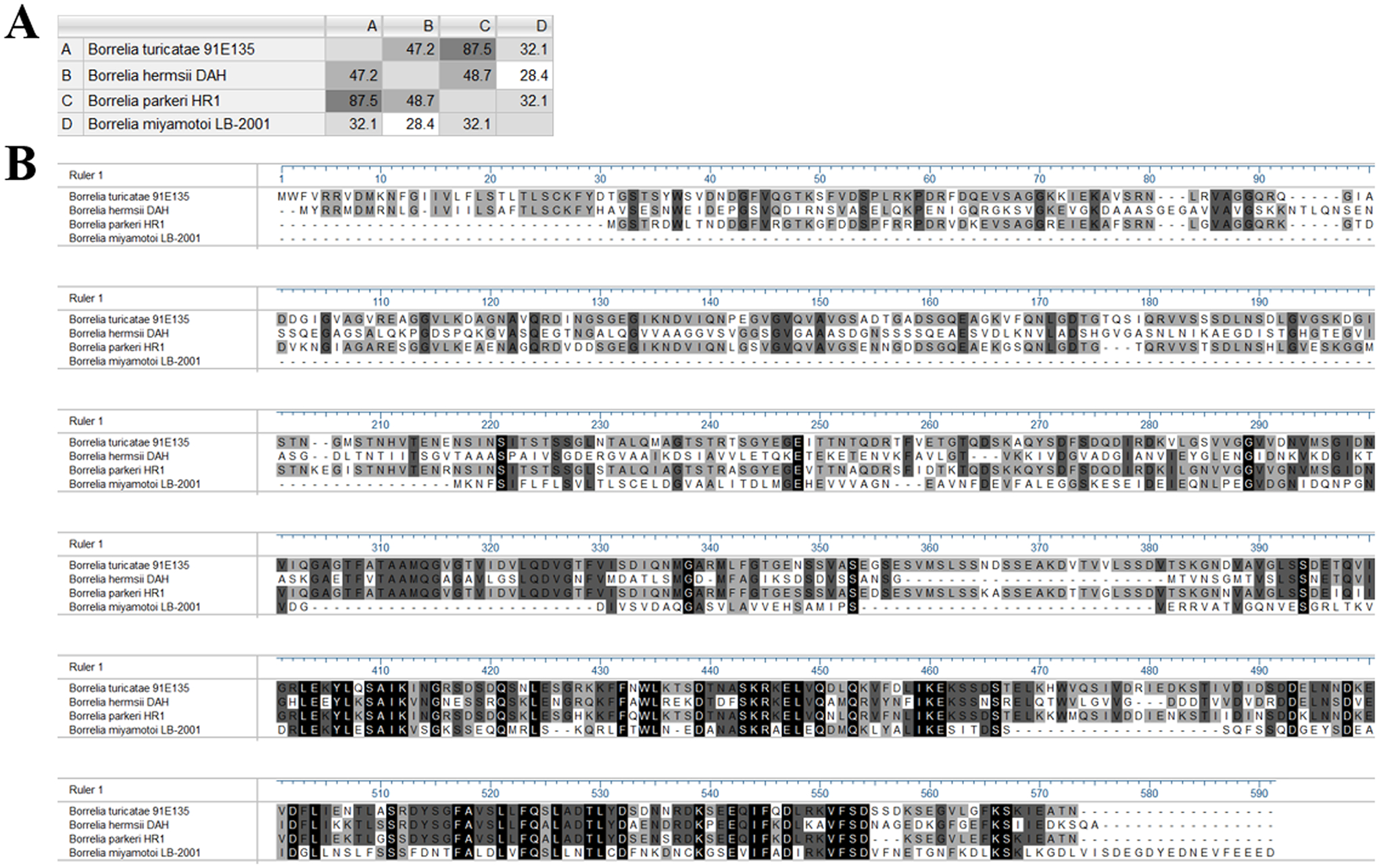
The percent identities of amino acid sequences for BipA proteins from *B. turicatae* 91E135 (row and column A), *B. hermsii* DAH (row and column B), *B. parkeri* HR1 (row and column C) compared with the sequence of the BipA homolog identified in *B. miyamotoi* LB-2001 (row and column D). (A) Alignment of BipA amino acid sequences from *B. turicatae* 91E135 (ADN26518.1), *B. parkeri* HR1 (AHF45615.1), *B. hermsii* DAH (ACS27065.1), and the BipA homolog identified in *B. miyamotoi* LB-2001 (WP_070401628.1) (B). Identical amino acids in the sequences of proteins from the four RF *Borrelia* species are highlighted by shading. The lightest shade of gray denotes identical amino acids between at least two species, the medium shade indicates identical amino acids between at least three species, and the darkest shade indicates amino acids shared between all four of the species shown above.

*In silico* analysis of the identified *B. miyamotoi* BipA homolog was performed prior to cloning and expression. The gene sequence for the hypothetical protein targeted to clone as a BipA homolog in *B. miyamotoi* LB-2001 was 100% identical to the gene sequence for this protein in the *B. miyamotoi* CT13–2396. The percent identity of the amino acid sequence of this BipA homolog in *B. miyamotoi* was 32.1% to both *B. turicatae* BipA and *B. parkeri* BipA, and 28.4% to *B. hermsii* BipA (Fig. 1). The *B. miyamotoi* BipA homolog and *B. turicatae* GlpQ predicted amino acid lengths were and 32 kDa and 40 kDa respectively. This predicted size for the recombinant *B. miyamotoi* BipA was much smaller than other BipA proteins (58–61 kDa) from soft tick RF *Borrelia*, which after expression were expected to be visualized near 60–75 kDa based on the recombinant protein observations in Curtis et al. (2022). A gap in the sequence at the N-terminus of the protein, in addition to signal peptide removal, contributed to the truncated overall size (Fig. 1).

**Fig. 2.**
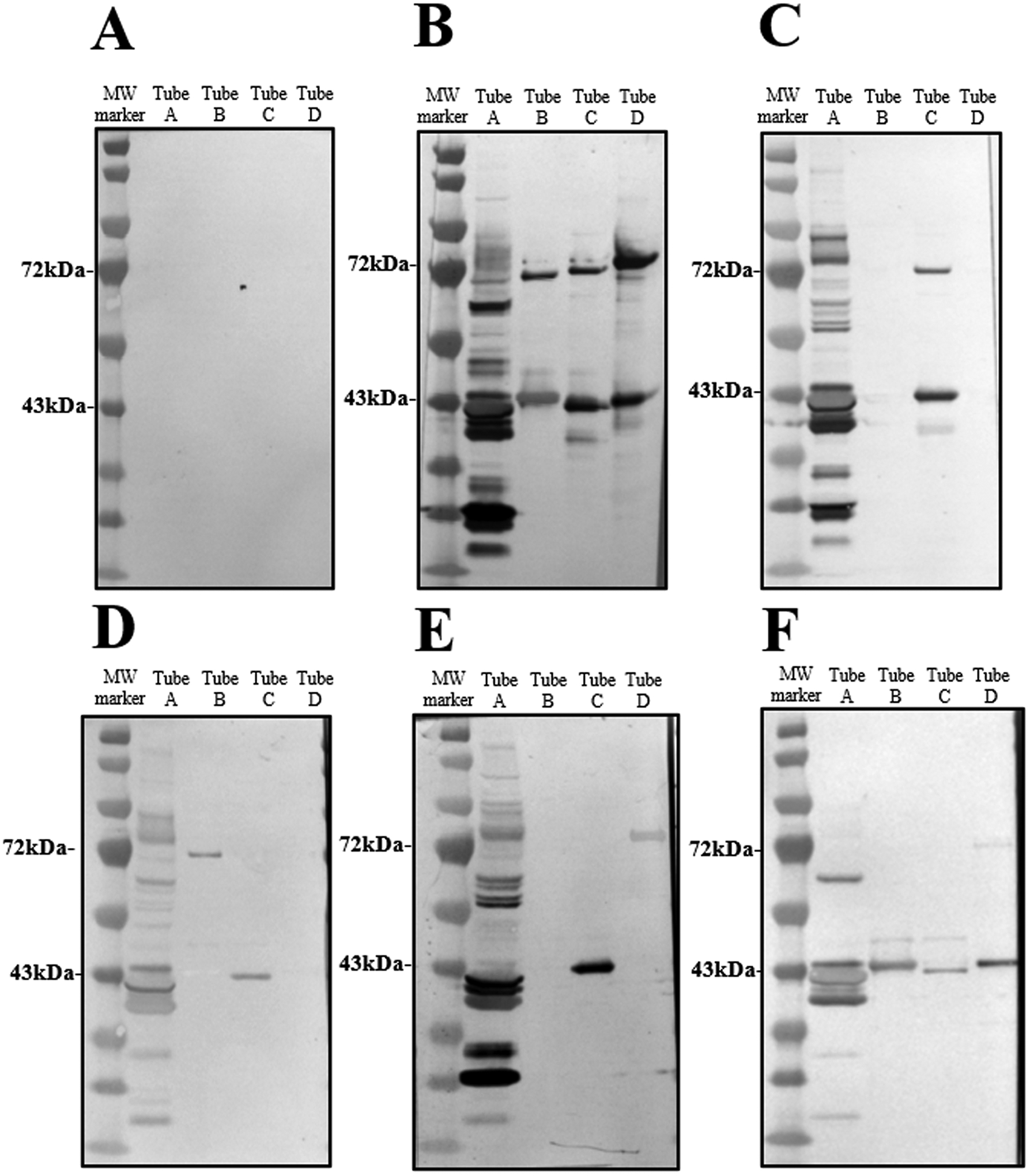
Serum from RF Borrelia exposed mice was tested with membranes containing Borrelia whole-cell lysate and recombinant antigen combinations. Tube A contained B. hermsii, B. parkeri, B. turicatae, and B. miyamotoi whole-cell lysates. Tubes B-D contained each of the following recombinant proteins: tube B: B. hermsii rBipA (72 kDa) and B. miyamotoi rBipA(43 kDa), tube C: B. parkeri rBipA (72 kDa) and rGlpQ (43 kDa), and tube D: B. turicatae rBipA (72 kDa) and rBmaA (43 kDa). Molecular weight is indicated in kilodaltons on the left of the blot. A negative control serum sample (A) and a positive control sample containing equal amounts of serum from individual mice infected with either B. hermsii, B. parkeri, B. turicate, or B. miyamotoi (B) were used to probe membranes each time samples were tested. Blots were probed with serum from individual mice infected with B. parkeri (C), B. hermsii (D), B. turicatae (E), and B. miyamotoi (F), were evaluated for reactivity with rBmaA and rGlpQ, and pathogen calls were made by the consensus of blinded raters based on the presence or absence of bands for species-specific rBipA targets.

Recombinant proteins were characterized post-expression in *E. coli* by gel electrophoresis, Coomassie staining, and Western blotting. Serum from a mouse inoculated with *B. miyamotoi* rBipA when used to probe the membrane indicated a strong band at 43 kDa, which was appearing larger after expression than the predicted molecular weight of the protein at 32 kDa (supplementary data). Additionally, the presence of bound antibodies suggested that the recombinant *B. miyamotoi* BipA *in vivo* can be antigenic. When the recombinant proteins were loaded individually, the rBipA targets for each of the soft tick RF *Borrelia* species were visualized according to the molecular weight marker used in this study at 72 kDa, and the rBipA from *B. miyamotoi*, rGlpQ, and rBmaA proteins at 43 kDa (supplementary data). Pools of recombinant antigens (tube B-D, Fig. 2) were formulated to include a protein target visualized at 72 kDa and at 43 kDa to ensure that there was enough separation by size amongst the six targets to identify each protein based off these results.

### 3.2. Agreement of sample call with expected results for whole-cell lysate and rBipA reactivity

Serum samples were evaluated for reactivity with the RF *Borrelia* whole-cell lysate and recombinant targets based on the expected results for each antigen listed in Table 3. Serum reactivity to the whole-cell lysate was the first criteria for samples to be assessed. Samples were eliminated from further testing if they failed to have five bands present in the tube A lane of the developed blot (negative control, blot A, Fig. 2). The exception to this was inclusion of negative control samples which were further evaluated throughout the assay to observe the background reactivity of the serum to the recombinant protein antigens used in this study. All mouse serum samples (*P. maniculatus* n= 3, *P. leucopus* n= 2, *M. musculus* n= 2) with no known *Borrelia* exposure history were non-reactive with all antigens tested for, having 100% agreement with expected results and all called negative (Tables 3 and 4). All serum samples from mice infected with *B. burgdorferi* s.s. (*M. musculus* n= 2) and *B. mayonii (P. leucopus* n= 3) were called positive for *Borrelia*, inconclusive for species identification (negative for recombinant RF targets) by blinded raters and had 100% agreement with expected results (Tables 3 and 4).

*Borrelia miyamotoi* infected mouse serum samples (*P. maniculatus* n= 1, *M. musculus* n= 14) had the highest percentage of agreement between expected and observed results based on rBipA at 93.3%, 14 out of 15 serum samples called positive for *B. miyamotoi*, and one sample called positive for *Borrelia* but inconclusive for species identification (Tables 3 and 4). Serum generated from mice (*P. maniculatus* n= 7, *M. musculus* n= 2) infected with *B. hermsii* had an agreement of 88.9% with expected results, 8 out of 9 samples called positive for *B. hermsii*, and one sample called positive for *Borrelia* but inconclusive for species identification (Tables 3 and 4). Serum samples from mice (*P. maniculatus* n= 3, *M. musculus n*= 2) infected with *B. parkeri* had an 80% agreement with expected results, 4 out of 5 samples called positive for *B. parkeri*, and one sample called positive for *Borrelia* but inconclusive for species identification (Tables 3 and 4). *Borrelia turicatae* infected mouse serum samples (*P. maniculatus* = 8, *M. musculus* = 2) had the lowest level of agreement with expected results at 20%, with 2 out of 10 samples called positive for *B. turicatae*, however most were called positive for *Borrelia* but inconclusive for species identification (Tables 3 and 4).

All mouse samples classified as soft tick RF *Borrelia* species (*B. hermsii, B. parkeri*, or *B. turicatae*) were correctly identified as exposed to soft tick *Borrelia* species; 14 out of 14 total soft tick RF exposed samples were called correctly (Table 3). Classification of samples as hard tick RF *Borrelia* that were actually infected with *B. miyamotoi* was also found to be 100%, with 14 out of 14 samples called correctly (Table 3). By using the rBipA targets, we detected antibodies from three mouse species indicative of prior *Borrelia* exposure and identified samples infected with *B. miyamotoi* apart from soft tick RF *Borrelia* species.

### 3.3. Individual sample reactivity with the whole-cell lysate, rBipA, rGlpQ, and rBmaA

No cross reactivity among recombinant BipA protein targets was observed in samples originating from *P. maniculatus* and *M. musculus* mice infected with either *B. hermsii* or *B. miyamotoi* (Table 4). All samples from mice infected with these *Borrelia* species had bands present for recombinant targets that were predicted (Table 4) for the species of exposure only (*B. hermsii*: positive for *B. hermsii* rBipA with rGlpQ, and *B. miyamotoi*: positive for *B. miyamotoi* rBipA with either or both rBmaA and rGlpQ). Additionally, no bands for any RF recombinant antigens were observed from *P. leucopus* and *P. maniculatus* mice infected with *B. mayonii* or *B. burgdorferi* s.s. For these species, at least five bands were indeed present on the whole-cell lysate, which resulted in a call of positive for *Borrelia* but inconclusive for species identification. This indicates that LD *Borrelia* infected mouse serum samples had no observed cross reactivity with the recombinant RF antigens used in this study but were reactive with RF whole-cell lysates. One *B. hermsii* infected sample (*P. maniculatus* 7) was called positive for *Borrelia* but inconclusive for species due to the absence of the band for *B. hermsii* rBipA required to be called positive for *B. hermsii*. One *B. miyamotoi* CT13–2396 infected sample (*P. maniculatus* 1) was called positive for *Borrelia* but inconclusive for species due to the absence of a required band for *B. miyamotoi* rBipA, however a band for rBmaA was present. Among *B. miyamotoi* infected mouse samples, all samples were called positive for *B. miyamotoi* rBipA and rBmaA when mice were infected with isolate CT15–0840 *via I. scapularis* bite (Table 4).

Intraspecies cross reactivity amongst recombinant RF BipA targets was observed for mice infected with *B. turicatae* and *B. parkeri*. Bands for *B. hermsii* rBipA (5/10), *B. parkeri* rBipA (4/10), and rBmaA (7/10) in addition to the predicted *B. turicatae* rBipA and rGlpQ targets were present for both *P. maniculatus* and *M. musculus* serum samples from mice infected with *B. turicatae* (Table 4). For one *M. musculus* sample exposed to *B. parkeri*, a band for rBmaA and *B. turicatae* rBipA (1/5) was present in addition to the predicted targets *B. parkeri* rBipA and rGlpQ (Table 4). The presence of bands for rBipA derived from multiple soft tick RF *Borrelia* species resulted in a call of *Borrelia* but inconclusive for species for most *B. turicatae* (8/10) infected mouse samples, and one *B. parkeri* (1/5) infected mouse sample (Table 4). No bands were present for *B. miyamotoi* rBipA (0/22) from samples originating from *B. hermsii, B. turicatae* or *B. parkeri* infected mice (Table 4).

## 4. Discussion

Due to the inconsistent dissemination of RF *Borrelia* in the blood of rodent hosts, and the occasional inability to obtain argasid ticks because of their nidicolous nature, direct detection methods employed to support environmental investigations of human RF cases are not always available to investigators. Tools such as serologic assays that detect prior exposure of an animal to RF spirochetes are often relied upon to detect pathogen presence in an area. Here, we described a serological assay based on reactivity with RF whole-cell lysates, three previously described recombinant BipA proteins (Curtis et al., 2022), and a newly synthesized recombinant BipA homolog from *B. miyamotoi* that together accurately differentiated soft tick from hard tick RF exposures. Segregation among soft tick RF species was inadequate owing to observed cross-reactivity among recombinant protein targets. This tool may be useful for environmental investigations when tick vectors are unable to be found, particularly in areas where both hard and soft tick associated RF *Borrelia* species overlap such as in the Pacific coast states (Xu et al., 2019; Fleshman et al., 2022).

To our knowledge, this is the first evaluation of hard tick vs. soft tick RF *Borrelia* exposed mouse serum with rBipA from various species of origin. The absence of reactivity of *B. miyamotoi* infected mouse serum with any of the soft tick RF derived recombinant rBipA proteins suggests that these BipA proteins are specific to only soft tick RF antibodies. Conversely, the absence of reactivity of the soft tick RF infected samples to *B. miyamotoi* rBipA indicates that this antigen could be used to distinguish hard tick vs. soft tick RF *Borrelia* exposure. This finding warrants further investigation into the specificity of this protein target. *Borrelia miyamotoi* infected mouse serum was found to have reactivity with rGlpQ originating from *B. turicatae*, yet this protein is known to be highly conserved across RF *Borrelia* species (Lopez et al., 2016), and was expected to detect each of the RF spirochete exposures. Furthermore, this work provides evidence to support the utility of rBmaA to separate RF and LD prior exposure in additional species of *Borrelia* infected mouse serum (*B. mayonii* and *Peromyscus* spp.), however evidence of cross-reactivity with *B. turicatae* and *B. parkeri* occurred. This indicates that reactivity with rBmaA is inadequate to separate hard tick from soft tick RF exposures.

One observation among the *B. miyamoto*i infected serum samples was that the mice inoculated *via* tick bite produced more serum samples reactive with the recombinant proteins when compared with mice exposed *via* needle inoculation. We used two isolates of *B. miyamotoi* to infect mice (CT13–2396 and CT15–0840), with the tick bite exposed mice (CT15–0840) having the more consistent results where the RF *Borrelia* whole-cell lysate and *B. miyamotoi* recombinant protein targets were reactive for all 13 samples. A mouse exposed *via* needle inoculation had reactivity only with the whole-cell lysate and one of the expected *B. miyamotoi* recombinant protein targets. This result could be due to differences between isolates used, or among proteins expressed when culture is artificially inoculated by needle versus natural inoculation by an infected tick vector. The role of the tick has been demonstrated to be extremely important in both soft tick and hard tick vectored transmission (Schwan et al., 2002; Nuttall et al., 2000; Barbour et al., 1986). Differences in recombinant antigen reactivity with antibodies from mice infected with *B. miyamotoi* by tick bite versus needle inoculation have been documented (Harris et al., 2019). Using tick-bite-infected mouse serum more closely replicates the natural enzootic cycle and is the ideal method for artificial exposure of mice to both LD and RF *Borrelia* species.

Although the assay performed well for discriminating among samples exposed to hard tick versus soft tick RF spirochetes, the assay was inadequately specific to accurately differentiate among soft tick RF species exposures. We observed a large amount of cross reactivity with rBipA among soft tick RF *Borrelia*, mostly by *B. turicatae* exposed mouse serum and *B. parkeri* infected mouse serum (Table 4). This result conflicts with the findings of Lopez et al. (2013) in which serum from animals infected with *B. turicatae* were non- reactive with *B. hermsii* rBipA. Inconsistent with previous observations of *B. turicatae* exposed human samples found to be non-reactive towards rBmaA (Harris et al., 2020), serum from mice infected with *B. turicatae* or *B. parkeri* in the current study reacted with this target (Table 4). Moreover, soft tick RF inter-species cross reactivity with rBipA has been previously noted from serum with *B. parkeri* and *B. turicatae* exposure history (Curtis et al., 2022). Shah et al. (2019) was also unable to distinguish between soft tick RF species of exposure using rBipA to classify samples. Our results suggests that mice infected with to *B. turicatae* can have some level of reactivity with *B. hermsii* and *B. parkeri* rBipAs (Table 4). This could result in a false positive for *B. hermsii* or *B. parkeri* exposure, and skepticism should be employed when interpreting results especially in an area where presence of a soft tick RF *Borrelia* species has not been previously documented. Recent studies describing the genome of *B. turicatae* (Kneubehl et al., 2022; Krishnavajhala et al., 2021) demonstrated that between isolates there is a high level of genetic variation. Using different isolates of RF *Borrelia* in the current study to infect mice could have contributed to differences in the levels of cross reactivity observed from previous work (Lopez et al., 2013; Curtis et al., 2022).

This work was not without limitations. There are notable differences between East and West coast *B. miyamotoi* genomes (Hojgaard et al., 2021; Cook et al., 2016), and more investigation is needed to evaluate if phenotypic differences in serologic reactivity occur. By using a West coast-derived *B. miyamotoi* isolate such as CA17–2241 from *I. pacificus* (Kingry et al., 2017a), potential cross reactivity among recombinant protein targets could be assessed. Another limitation of this study is that artificially infected mouse serum was used, rather than field-derived serum. Serum reactivity with *B. miyamotoi* rBipA derived from other species apart from rodents remains unconfirmed, and would be required to evaluate this assay for use with field derived serum with unknown *Borrelia* exposure.

We have tested an assay designed to evaluate animal samples for reactivity with RF *Borrelia* whole-cell lysates in addition to four recombinant RF *Borrelia* antigen targets to separate the presence of hard tick from soft tick RF and LD *Borrelia* exposure. We demonstrated the ability of these protein targets to react with laboratory derived serum from three mouse species infected with *Borrelia* spirochetes. The ability to detect and differentiate hard and soft tick RF *Borrelia* exposure was observed from reactivity with the rBipA targets, although the ability to classify exposure to a *Borrelia* species was lacking among soft tick RF exposed serum samples. With further evaluation using field collected samples derived from a variety of host species, this serologic testing algorithm could be useful to identify an area of potential human exposure to RF *Borrelia*.

## Supplementary Material

supplementary data

## Figures and Tables

**Table 1:** Primers used to amplify recombinant protein genes.

Primers	Sequence (5′–3′)	Ref.
*B. turicatae glpQ* -F	CATCATCACCACCATCACAAATYAATTAAAVCAAAATYA	This study
*B. turicatae glpQ* -R	GTGGCGGCCGCTCTATTATTATTGTTTTACAAACTTCAC	This study
*B. miyamotoi bipA* -F	CATCATCACCACCATCACGAACTTGATGGTGTAGCCGCA	This study
*B. miyamotoi bipA* -R	GTGGCGGCCGCTCTATTAATCTTCTTCTTCAAAAACTTC	This study
*B. miyamotoi bmaA* -F	CATCATCACCACCATCACAGTAATTTGATATCTCAGGAG	Harris et al. (2019)
*B. miyamotoi bmaA* -R	GTGGCGGCCGCTCTATTATCTTTCAAGAGTCCTCTAAAC	Harris et al. (2019)

**Table 2:** Serum samples included in this study from rodents challenged with various *Borrelia* spp.

Rodent species	*Borrelia* spp.	No. of wks. after exposure blood was collected	No. of samples included	Exposure method	Sample type/ infection confirmation method	Ref.
*P. maniculatus*	*B. hermsii*	12	7	Needle inoculation	Blood/ PCR	This study
	*B. parkeri*	12	3	Needle inoculation	Blood/ PCR	This study
	*B. turicatae*	12	8	Needle inoculation	Blood/ PCR	This study
	*B. miyamotoi* (CT13-2396)	12	1	Needle inoculation	Blood/ PCR	This study
	none	NA	3	NA	NA	This study
*P. leucopus*	none	NA	2	NA	NA	Parise et al. (2020)
	*B. mayonii*	12	3	Tick bite	Ear tissue/ culture	Parise et al. (2020)
*M. musculus*	*B. hermsii*	12	2	Needle inoculation	Blood/ PCR	This study
	*B. parkeri*	12	2	Needle inoculation	Blood/ PCR	This study
	*B. turicatae*	12	2	Needle inoculation	Blood/ PCR	This study
	*B. miyamotoi* (CT13-2396)	12	1	Needle inoculation	Blood/ PCR	This study
	*B. miyamotoi* (CT15-0840)	8–10	13	Tick bite	Blood/ PCR	Breuner et al. (2017)
	*B. burgdorferi* s.s.	4	2	Tick bite	Ear tissue/ culture	Breuner et al. (2020)
	none	NA	2	NA	NA	This study
	Total	51				

**Table 3:** Expected reactivity of target antigens based on exposure.

*Borrelia* spp. to which mice are exposed	Expected results					Observed results	
	RF *Borrelia* whole-cell lysate	*B. hermsii* rBipA	*B. miyamotoi* rBipA	*B. parkeri* rBipA	*B. turicatae* rBipA	Ratio of samples aligning with expected results/ total samples (percentage)	Ratio of samples classified as soft tick RF vs. hard tick RF *Borrelia*
*B. hermsii*	+	+	−	−	−	8/9 (88.9%)	14/14 classified as soft tick RF spp. *Borrelia*
*B. parkeri*	+	−	−	+	−	4/5 (80%)	
*B. turicatae*	+	−	−	−	+	2/10 (20%)	
*B. miyamotoi*	+	−	+	−	−	14/15 (93.3%)	14/14 classified as hard tick RF spp. *Borrelia*
*B. mayonii*	+/−^a^	−	−	−	−	3/3 (100%)	NA
*B. burgdorferi* s.s.	+/−^a^	−	−	−	−	2/2 (100%)	
none	−	−	−	−	−	7/7 (100%)	

a Indicates that LD *Borrelia* exposed serum samples can either be positive or negative on the whole-cell lysate without interfering with expected results for pathogen calls.

**Table 4:** Whole-cell lysate and recombinant protein target reactivity with serum samples from mice challenged with various *Borrelia* spp.

*Borrelia* spp.	Rodent species/ ID	RF *Borrelia* whole-cell lysate	*B. hermsii* rBipA	*B. miyamotoi* rBipA	*B. parkeri* rBipA	*B. turicatae* rBipA	rGlpQ	rBmaA	Final call
*B. hermsii*	*P. maniculatus* 1	+	+	−	−	−	+	−	*B. hermsii*
	*P. maniculatus* 2	+	+	−	−	−	+	−	*B. hermsii*
	*P. maniculatus* 3	+	+	−	−	−	+	−	*B. hermsii*
	*P. maniculatus* 4	+	+	−	−	−	+	−	*B. hermsii*
	*P. maniculatus* 5	+	+	−	−	−	+	−	*B. hermsii*
	*P. maniculatus* 6	+	+	−	−	−	+	−	*B. hermsii*
	*P. maniculatus* 7	+	−	−	−	−	+	−	*Borrelia*, inconclusive
	*M. musculus* 1	+	+	−	−	−	+	−	*B. hermsii*
	*M. musculus* 2	+	+	−	−	−	+	−	*B. hermsii*
*B. parkeri*	*P. maniculatus* 1	+	−	−	+	−	+	−	*B. parkeri*
	*P. maniculatus* 2	+	−	−	+	−	+	−	*B. parkeri*
	*P. maniculatus* 3	+	−	−	+	−	+	−	*B. parkeri*
	*M. musculus* 1	+	−	−	+	+	+	+	*Borrelia*, inconclusive
	*M. musculus* 2	+	−	−	+	−	+	−	*B. parkeri*
*B. turicatae*	*P. maniculatus* 1	+	−	−	−	+	+	−	*B. turicatae*
	*P. maniculatus* 2	+	−	−	−	+	+	+	*B. turicatae*
	*P. maniculatus* 3	+	+	−	−	+	+	+	*Borrelia*, inconclusive
	*P. maniculatus* 4	+	−	−	+	+	+	+	*Borrelia*, inconclusive
	*P. maniculatus* 5	+	+	−	−	+	+	+	*Borrelia*, inconclusive
	*P. maniculatus* 6	+	+	−	−	+	+	+	*Borrelia*, inconclusive
	*P. maniculatus* 7	+	+	−	+	+	+	+	*Borrelia*, inconclusive
	*P. maniculatus* 8	+	+	−	−	+	+	+	*Borrelia*, inconclusive
	*M. musculus* 1	+	−	−	+	+	+	−	*Borrelia*, inconclusive
	*M. musculus* 2	+	−	−	+	+	+	−	*Borrelia*, inconclusive
*B. miyamotoi* (CT13-2396)	*P. maniculatus* 1	+	−	−	−	−	−	+	*Borrelia, inconclusive*
	*M. musculus* 1	+	−	+	−	−	−	+	*B. miyamotoi*
*B. miyamotoi* (CT15-0840)	*M. musculus* 1	+	−	+	−	−	+	+	*B. miyamotoi*
	*M. musculus* 2	+	−	+	−	−	+	+	*B. miyamotoi*
	*M. musculus* 3	+	−	+	−	−	−	+	*B. miyamotoi*
	*M. musculus* 4	+	−	+	−	−	+	+	*B. miyamotoi*
	*M. musculus* 5	+	−	+	−	−	+	+	*B. miyamotoi*
	*M. musculus* 6	+	−	+	−	−	−	+	*B. miyamotoi*
	*M. musculus* 7	+	−	+	−	−	+	+	*B. miyamotoi*
	*M. musculus* 8	+	−	+	−	−	+	+	*B. miyamotoi*
	*M. musculus* 9	+	−	+	−	−	−	+	*B. miyamotoi*
	*M. musculus* 10	+	−	+	−	−	+	+	*B. miyamotoi*
	*M. musculus* 11	+	−	+	−	−	+	+	*B. miyamotoi*
	*M. musculus* 12	+	−	+	−	−	+	+	*B. miyamotoi*
	*M. musculus* 13	+	−	+	−	−	+	+	*B. miyamotoi*
none	*P. maniculatus* 1	−	−	−	−	−	−	−	negative
	*P. maniculatus* 2	−	−	−	−	−	−	−	negative
	*P. maniculatus* 3	−	−	−	−	−	−	−	negative
	*P. leucopus* 1	−	−	−	−	−	−	−	negative
	*P. leucopus* 2	−	−	−	−	−	−	−	negative
	*M. musculus* 1	−	−	−	−	−	−	−	negative
	*M. musculus* 2	−	−	−	−	−	−	−	negative
*B. mayonii*	*P. leucopus* 1	+	−	−	−	−	−	−	*Borrelia, inconclusive*
	*P. leucopus* 2	+	−	−	−	−	−	−	*Borrelia, inconclusive*
	*P. leucopus* 3	+	−	−	−	−	−	−	*Borrelia, inconclusive*
*B. burgdorferi* s.s.	*M. musculus* 1	+	−	−	−	−	−	−	*Borrelia, inconclusive*
	*M. musculus* 2	+	−	−	−	−	−	−	*Borrelia, inconclusive*

## Data Availability

Data will be made available on request.
