# Supplementary material for: A serological assay to detect and differentiate rodent exposure to soft tick and hard tick relapsing fever infections in the United States
Source: Ticks Tick Borne Dis. Author manuscript; Available in PMC 2024 Mar 21. (PMC10956445; doi:10.1016/j.ttbdis.2023.102167)
Supplement: supplementary data [file NIHMS1973396-supplement-supplementary_data.pdf]

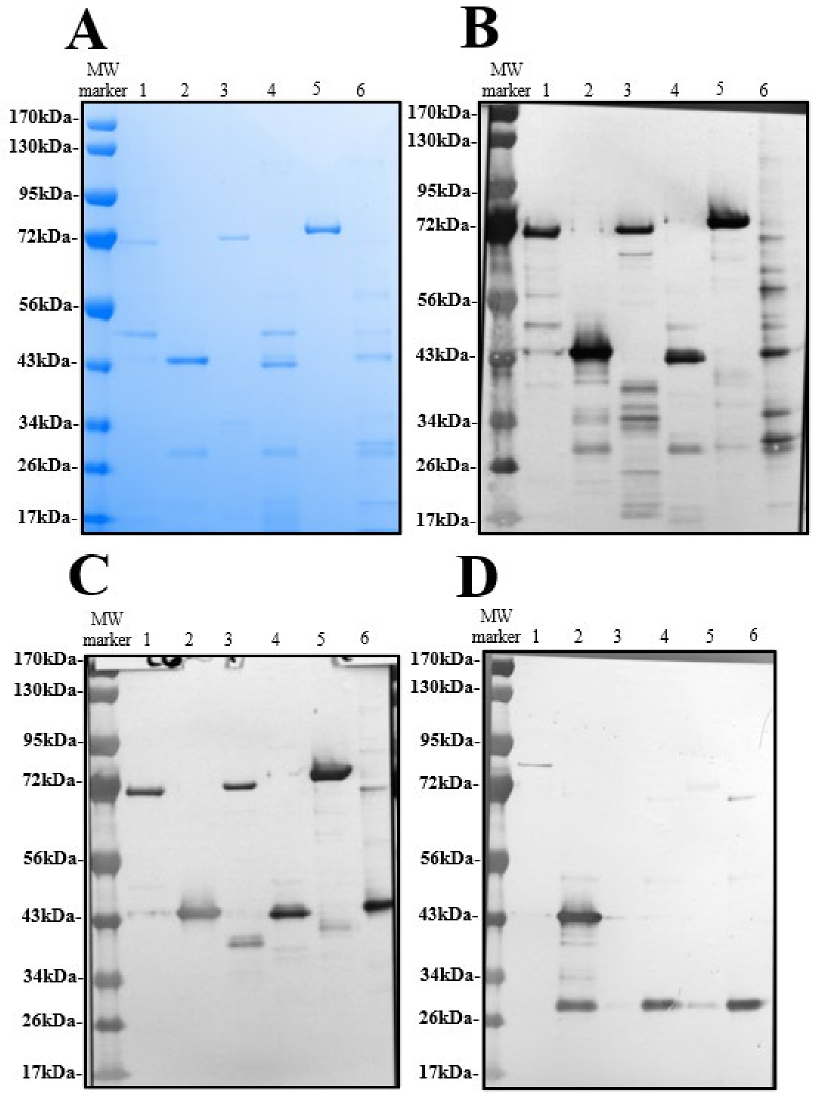

Supplementary Data. Characterization of recombinant protein targets by gel electrophoresis and western blotting. Recombinant targets were loaded for all gels into lanes 1-6 as follows: *B. hermsii* rBipA (lane 1), *B. miyamotoi* rBipA (lane 2), *B. parkeri* rBipA (lane 3), rGlpQ (lane 4), *B. turicatae* rBipA (lane 5), rBmaA (lane 6). Molecular weight is indicated in kilodaltons on the left of the blot. Proteins were electrophoresed and Coomassie stained (A) or transferred to membranes and probed with a x6 His monoclonal antibody (B), a positive control with equal amounts of serum from individual mice infected with either *B. miyamotoi, B. hermsii, B. parkeri*, or *B. turicatae* (C), or serum from a mouse immunized with *B. miyamotoi* rBipA (D).
